# Supplementary figures and images for: SRSF10 is essential for progenitor spermatogonia expansion by regulating alternative splicing
Source: eLife. 2022 Nov 10;11:e78211. doi: 10.7554/eLife.78211 (PMC9648972; doi:10.7554/eLife.78211)

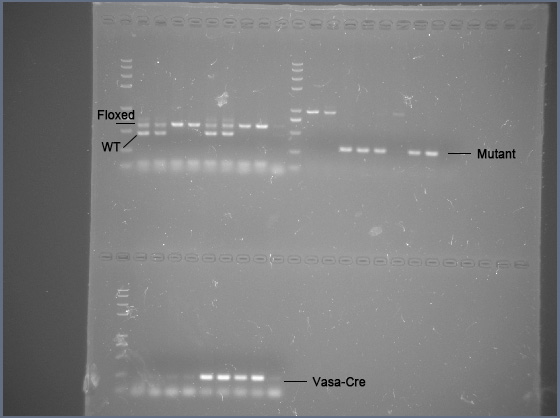

Supplement: Figure 1—figure supplement 1—source data 1. [file elife-78211-fig1-figsupp1-data1.zip › Figure 1-figure supplement 1-source data 1/Figure 1-figure supplement 1-labeled gel.jpg]

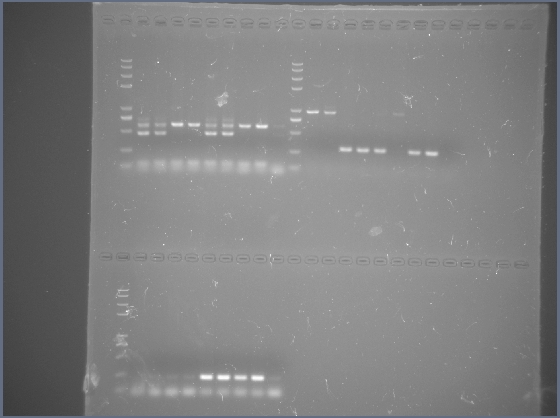

Supplement: Figure 1—figure supplement 1—source data 1. [file elife-78211-fig1-figsupp1-data1.zip › Figure 1-figure supplement 1-source data 1/Figure 1-figure supplement 1-uncropped and unedited gel.jpg]

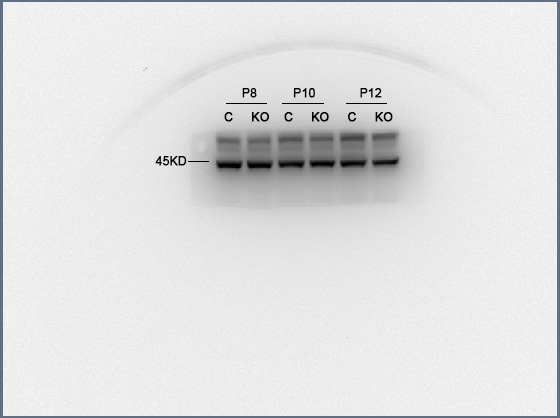

Supplement: Figure 2—source data 2. [file elife-78211-fig2-data2.zip › Figure 2-source data 2/Actin-labeled blot.jpg]

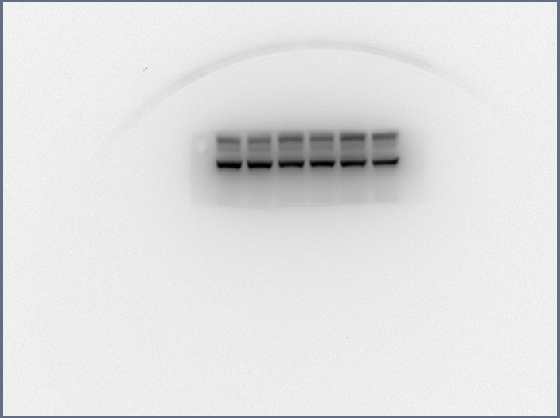

Supplement: Figure 2—source data 2. [file elife-78211-fig2-data2.zip › Figure 2-source data 2/Actin-uncropped unedited blot.jpg]

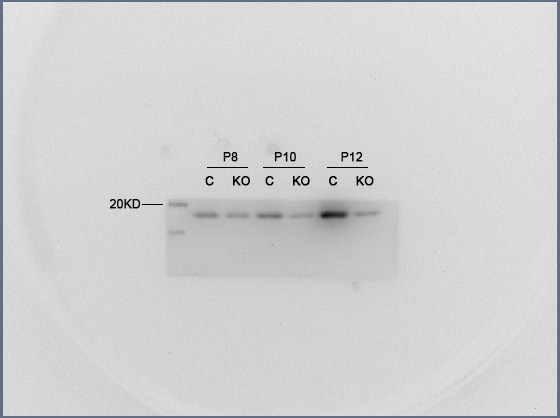

Supplement: Figure 2—source data 2. [file elife-78211-fig2-data2.zip › Figure 2-source data 2/gH2AX-labeled blot.jpg]

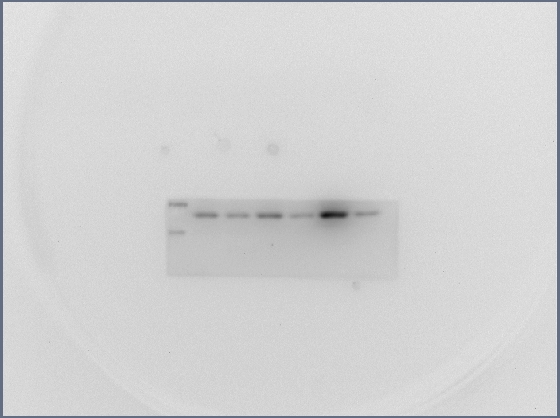

Supplement: Figure 2—source data 2. [file elife-78211-fig2-data2.zip › Figure 2-source data 2/gH2AX-uncropped and unedited blot.jpg]

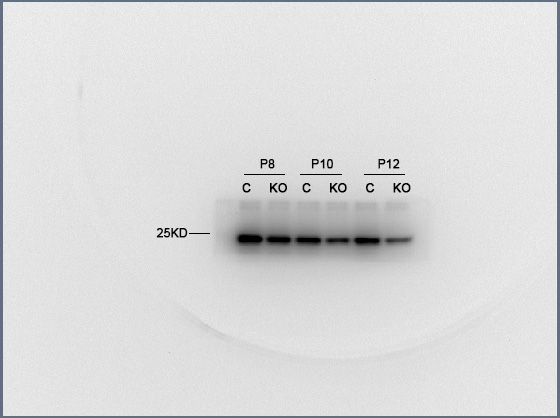

Supplement: Figure 2—source data 2. [file elife-78211-fig2-data2.zip › Figure 2-source data 2/SRSF10-labeled blot.jpg]

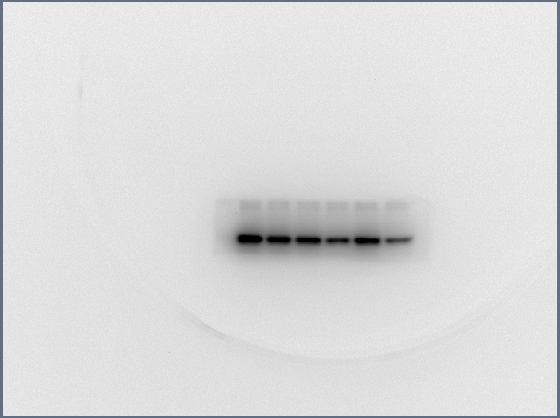

Supplement: Figure 2—source data 2. [file elife-78211-fig2-data2.zip › Figure 2-source data 2/SRSF10-uncropped and unedited blot.jpg]

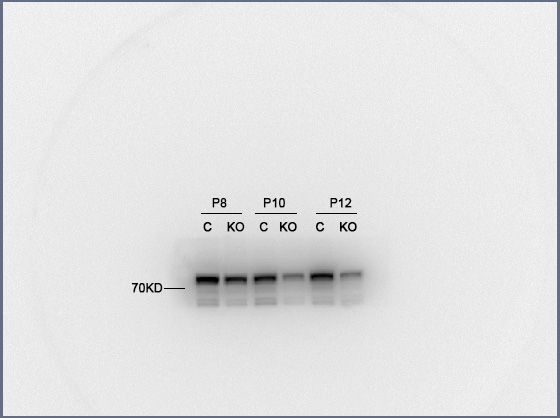

Supplement: Figure 3—source data 1. [file elife-78211-fig3-data1.zip › Figure 3-source data 1/MVH-labeled blot.jpg]

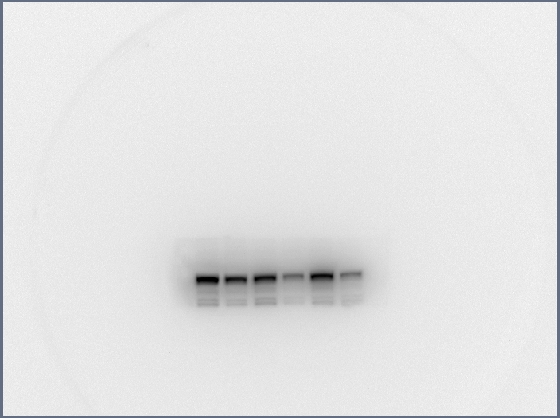

Supplement: Figure 3—source data 1. [file elife-78211-fig3-data1.zip › Figure 3-source data 1/MVH-uncropped and unedited blot.jpg]

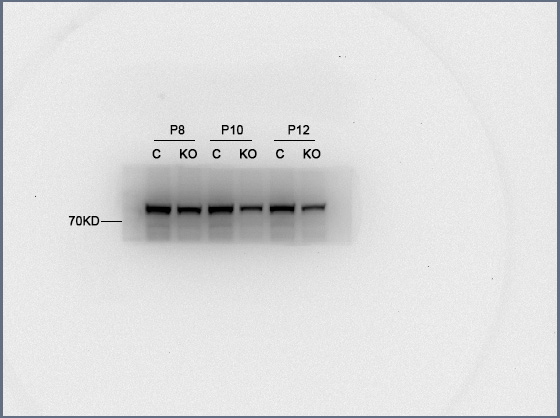

Supplement: Figure 3—source data 1. [file elife-78211-fig3-data1.zip › Figure 3-source data 1/PLZF-labeled blot.jpg]

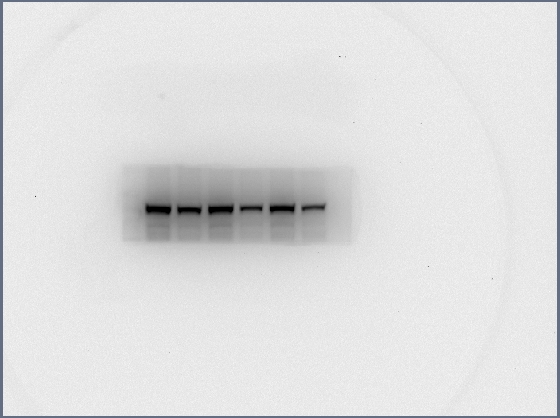

Supplement: Figure 3—source data 1. [file elife-78211-fig3-data1.zip › Figure 3-source data 1/PLZF-uncropped and unedited blot.jpg]

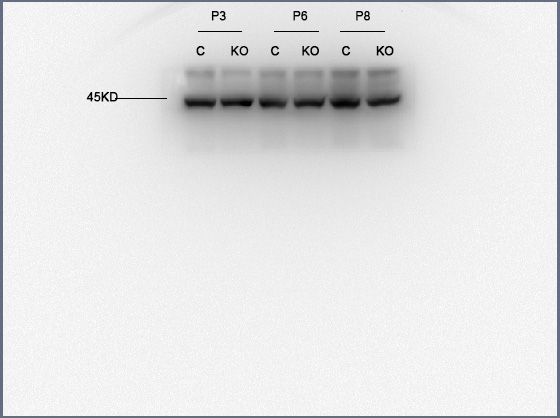

Supplement: Figure 3—figure supplement 1—source data 2. [file elife-78211-fig3-figsupp1-data2.zip › Figure 3-figure supplement 1-source data 2/Actin-labeled blot.jpg]

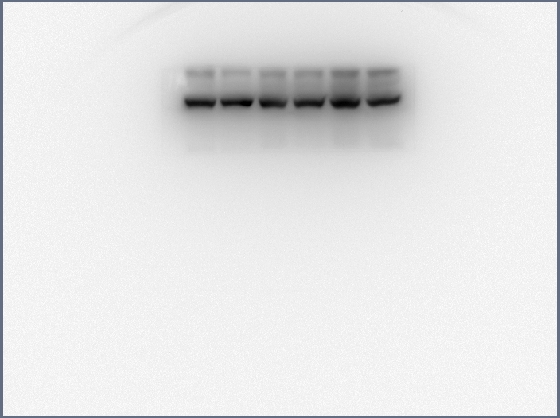

Supplement: Figure 3—figure supplement 1—source data 2. [file elife-78211-fig3-figsupp1-data2.zip › Figure 3-figure supplement 1-source data 2/Actin-uncropped and unedited blot.jpg]

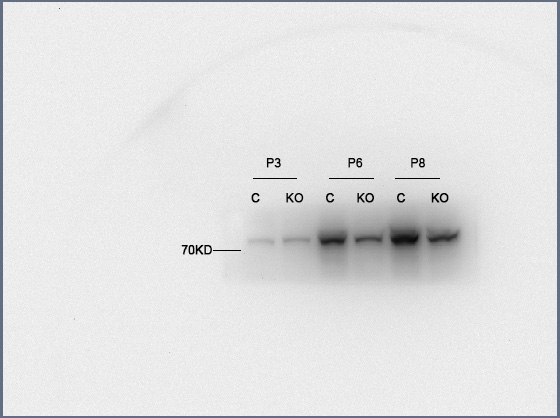

Supplement: Figure 3—figure supplement 1—source data 2. [file elife-78211-fig3-figsupp1-data2.zip › Figure 3-figure supplement 1-source data 2/PLZF-labeled blot.jpg]

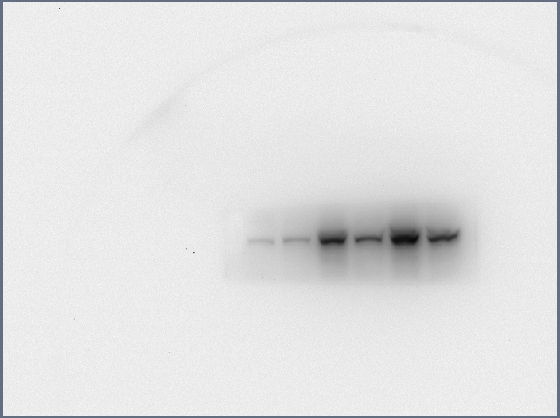

Supplement: Figure 3—figure supplement 1—source data 2. [file elife-78211-fig3-figsupp1-data2.zip › Figure 3-figure supplement 1-source data 2/PLZF-uncropped and unedited blot.jpg]

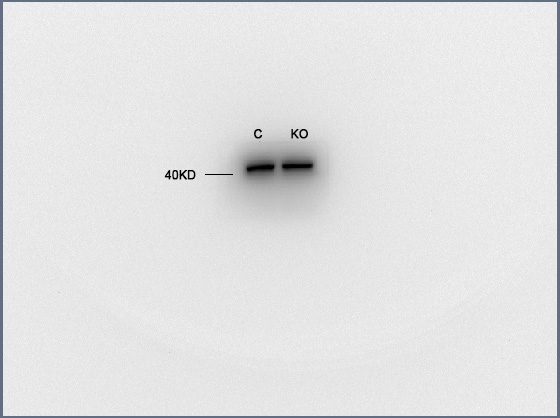

Supplement: Figure 7—figure supplement 1—source data 1. [file elife-78211-fig7-figsupp1-data1.zip › Figure 7-figure supplement 1-source data 1/Figure 7- figure supplement 1-Blot source data/Actin-labeled blot.jpg]

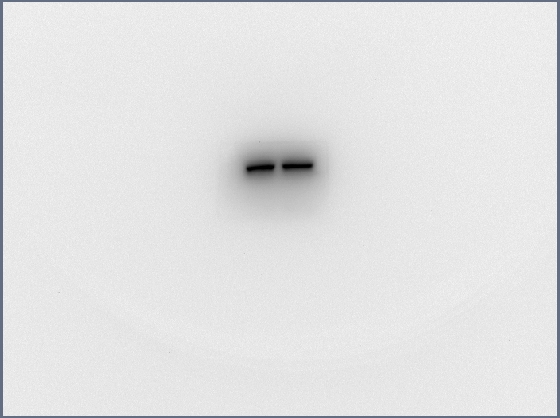

Supplement: Figure 7—figure supplement 1—source data 1. [file elife-78211-fig7-figsupp1-data1.zip › Figure 7-figure supplement 1-source data 1/Figure 7- figure supplement 1-Blot source data/Actin-uncropped and unedited blot.jpg]

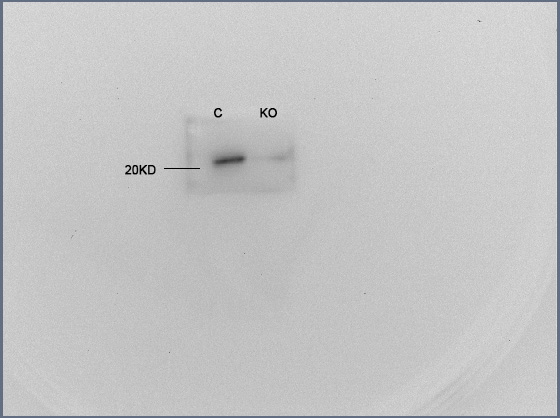

Supplement: Figure 7—figure supplement 1—source data 1. [file elife-78211-fig7-figsupp1-data1.zip › Figure 7-figure supplement 1-source data 1/Figure 7- figure supplement 1-Blot source data/SRSF10-labeled blot.jpg]

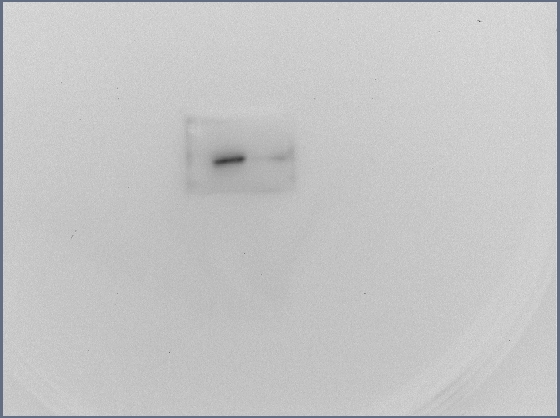

Supplement: Figure 7—figure supplement 1—source data 1. [file elife-78211-fig7-figsupp1-data1.zip › Figure 7-figure supplement 1-source data 1/Figure 7- figure supplement 1-Blot source data/SRSF10-uncropped and unedited blot.jpg]

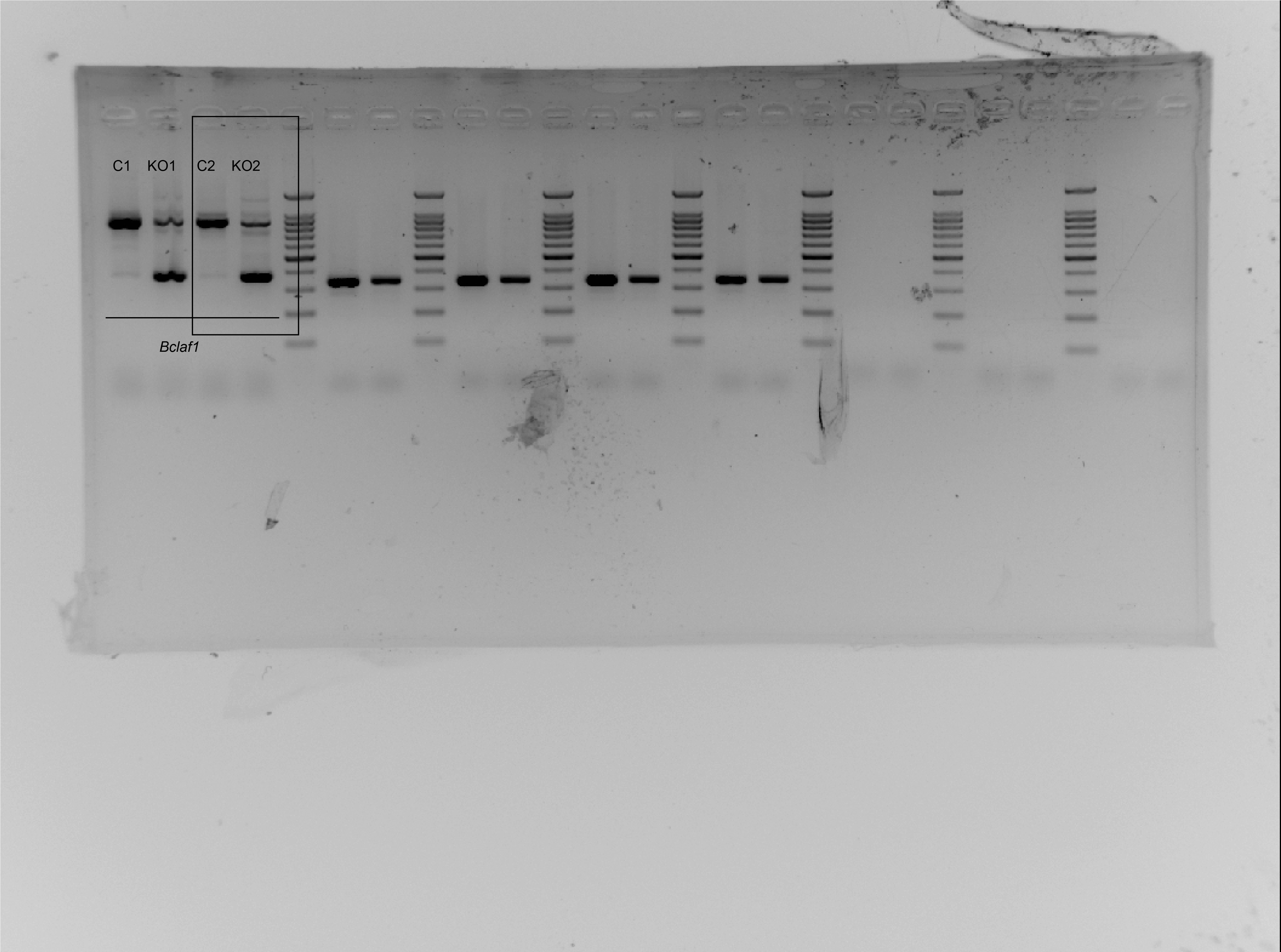

Supplement: Figure 8—source data 3. [file elife-78211-fig8-data3.zip › Figure 8-source data 3/Bclaf1-labeled gel for Figure 8D.jpg]

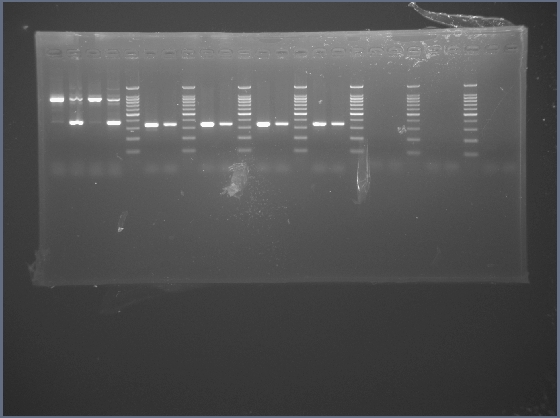

Supplement: Figure 8—source data 3. [file elife-78211-fig8-data3.zip › Figure 8-source data 3/Bclaf1-uncropped and unedited gel.jpg]

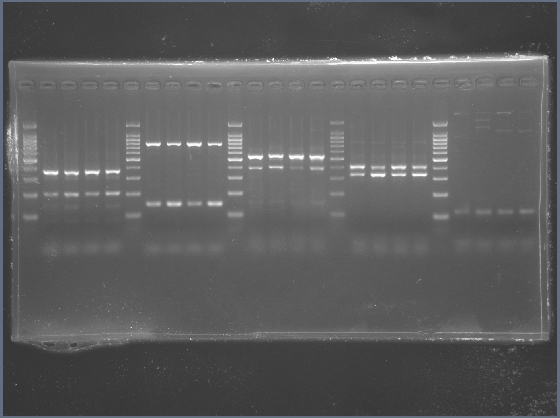

Supplement: Figure 8—source data 3. [file elife-78211-fig8-data3.zip › Figure 8-source data 3/Cenph Mcm10 and Rif1-uncropped and unedited gel.jpg]

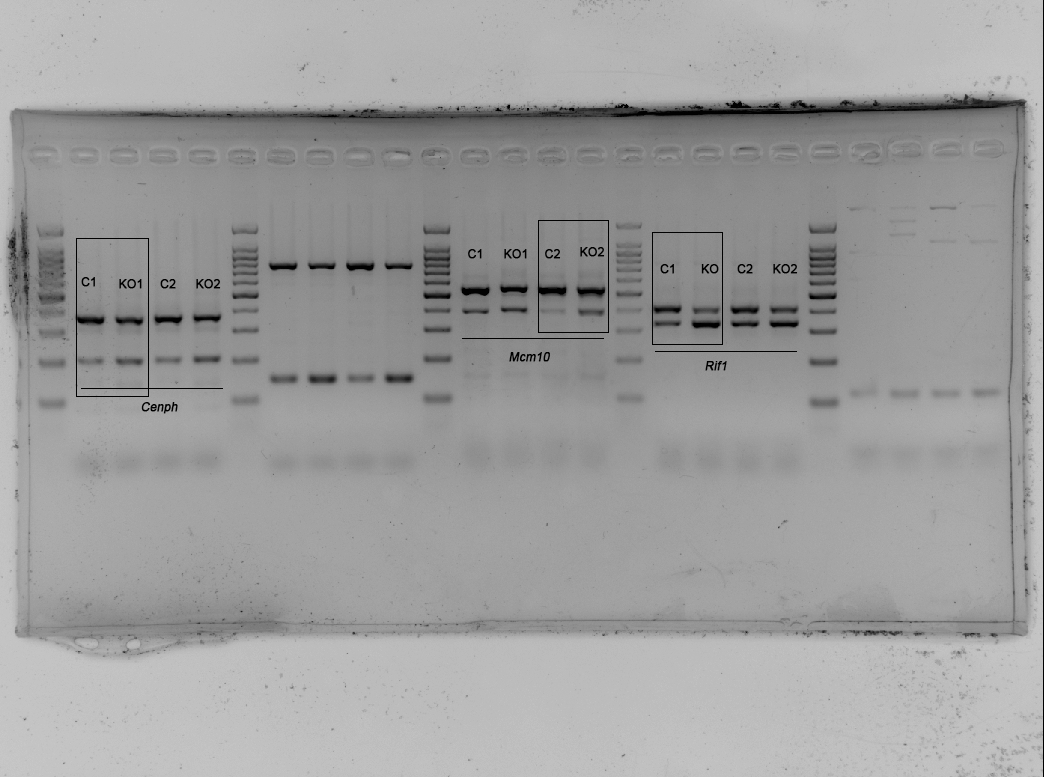

Supplement: Figure 8—source data 3. [file elife-78211-fig8-data3.zip › Figure 8-source data 3/Cenph Mcm10 Rif1-labeled gel for Figure 8D.tif]

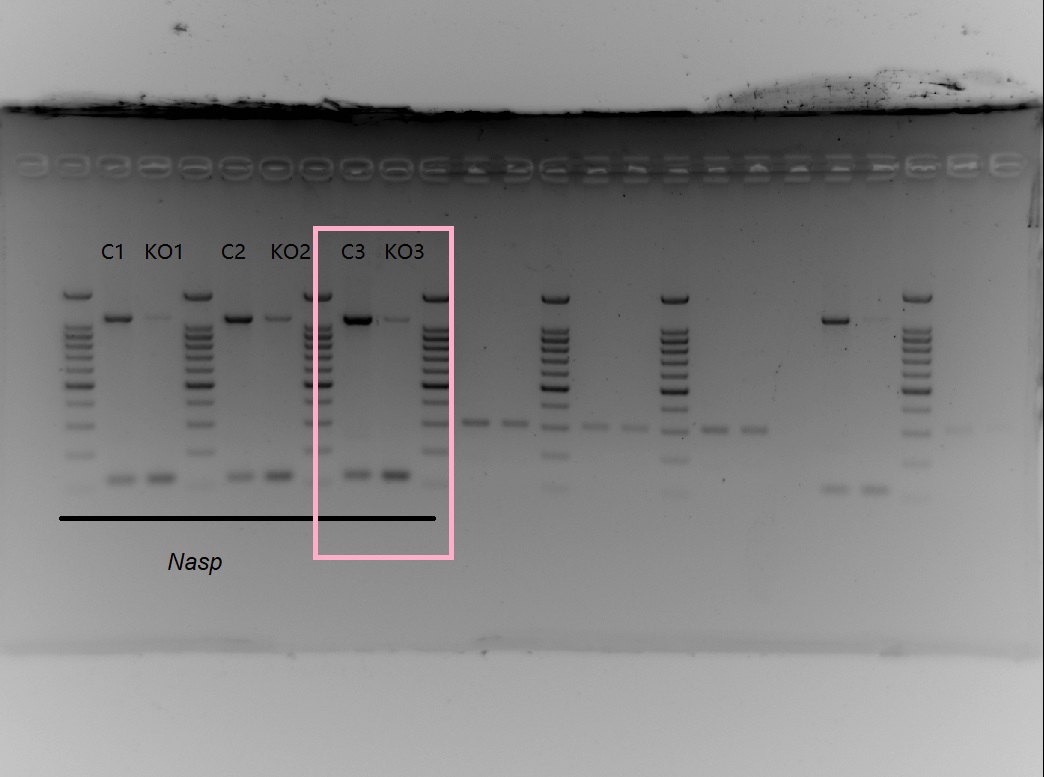

Supplement: Figure 8—source data 3. [file elife-78211-fig8-data3.zip › Figure 8-source data 3/Nasp-labeled gel for Figure 8D.jpg]

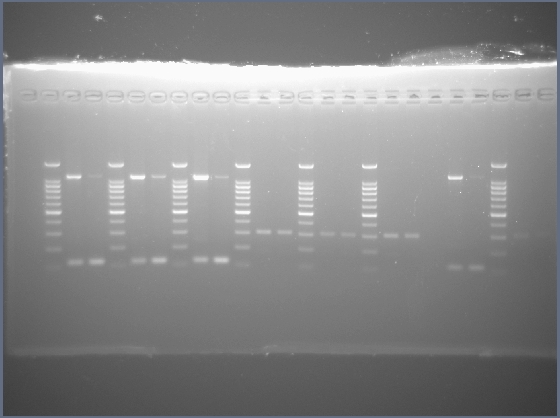

Supplement: Figure 8—source data 3. [file elife-78211-fig8-data3.zip › Figure 8-source data 3/Nasp-uncropped and unedited gel.jpg]

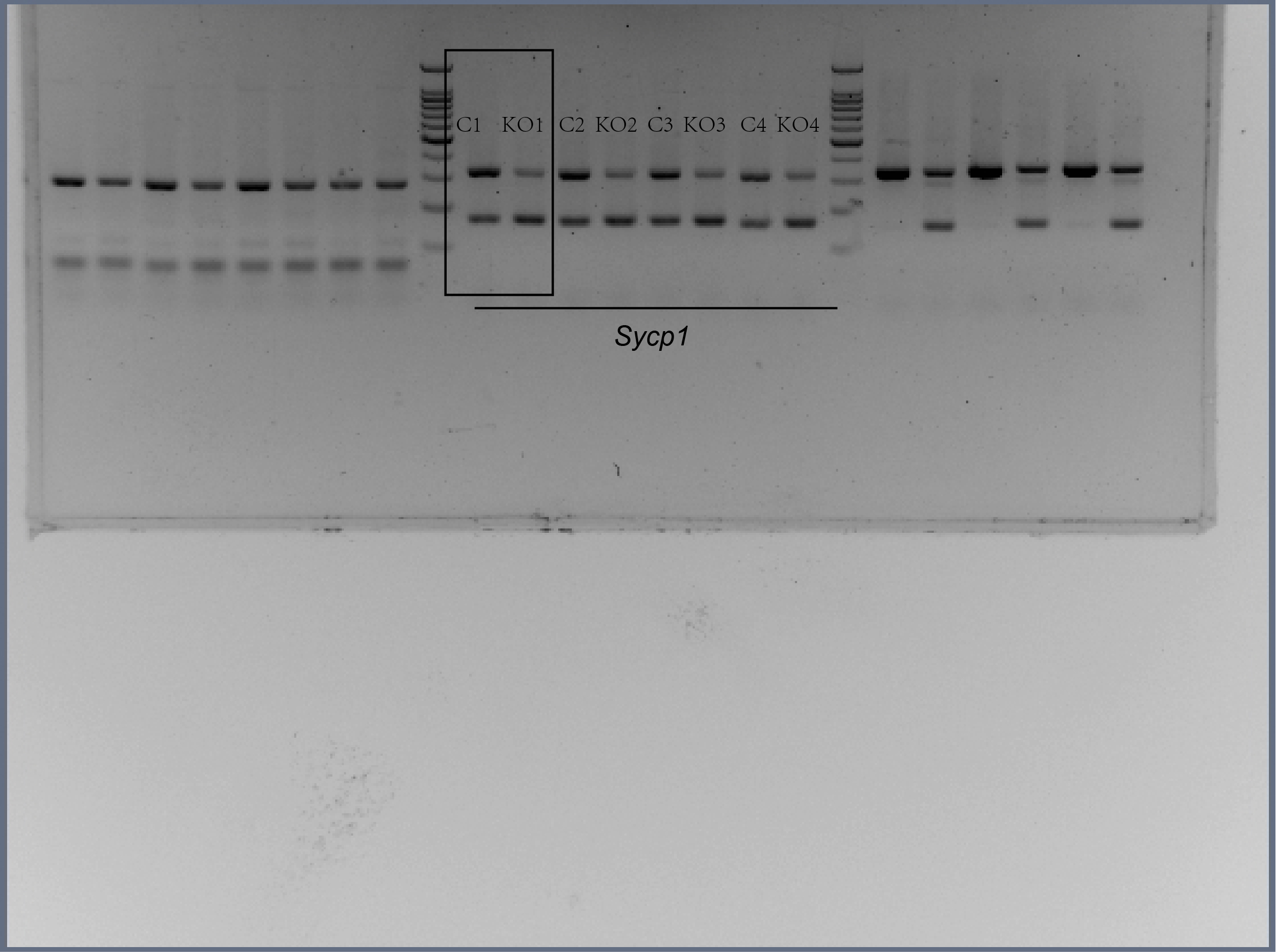

Supplement: Figure 8—source data 3. [file elife-78211-fig8-data3.zip › Figure 8-source data 3/Sycp1-labeled gel for Figure 8D.jpg]

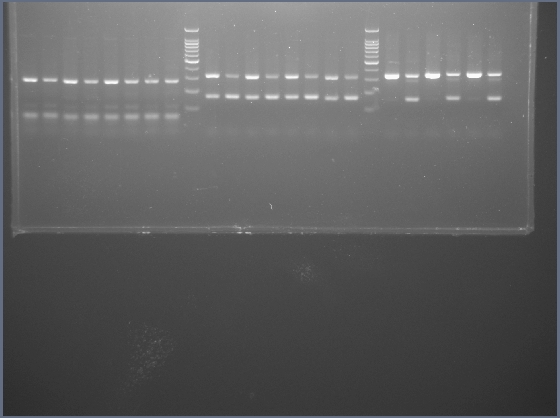

Supplement: Figure 8—source data 3. [file elife-78211-fig8-data3.zip › Figure 8-source data 3/Sycp1-uncropped and unedited gel.jpg]

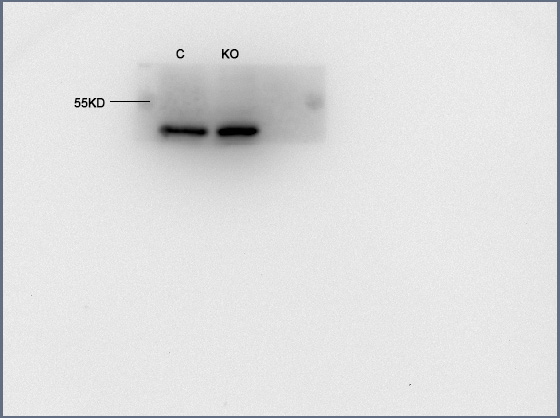

Supplement: Figure 8—source data 4. [file elife-78211-fig8-data4.zip › Figure 8-source data 4/BCLAF1/Actin-labeled blot.jpg]

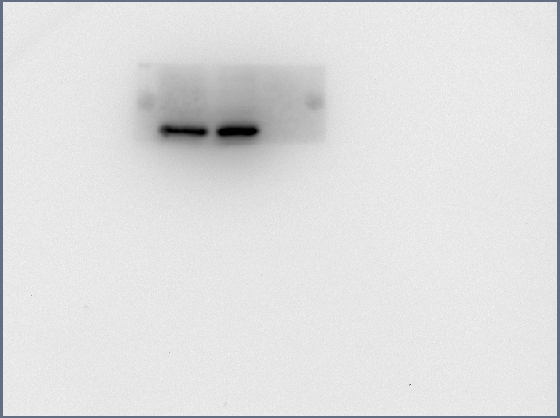

Supplement: Figure 8—source data 4. [file elife-78211-fig8-data4.zip › Figure 8-source data 4/BCLAF1/Actin-uncropped and unedited blot.jpg]

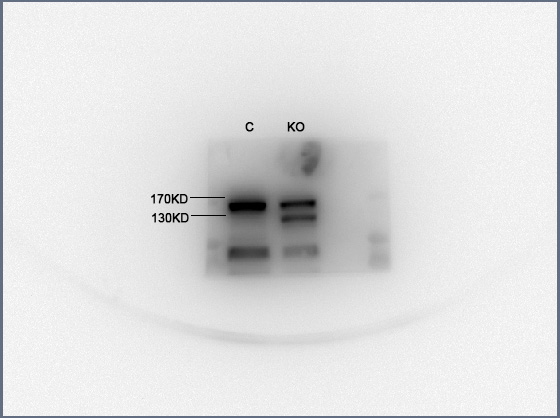

Supplement: Figure 8—source data 4. [file elife-78211-fig8-data4.zip › Figure 8-source data 4/BCLAF1/BCLAF1-labeled blot.jpg]

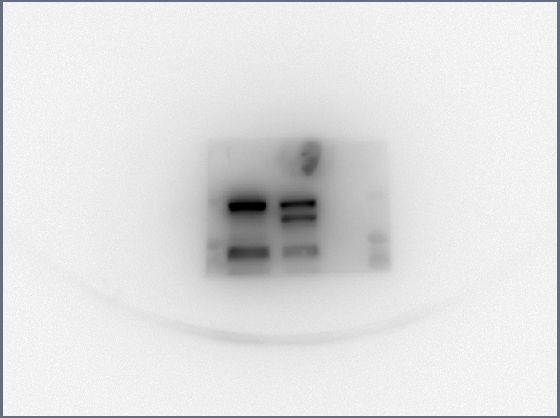

Supplement: Figure 8—source data 4. [file elife-78211-fig8-data4.zip › Figure 8-source data 4/BCLAF1/BCLAF1-uncropped and unedited.jpg]

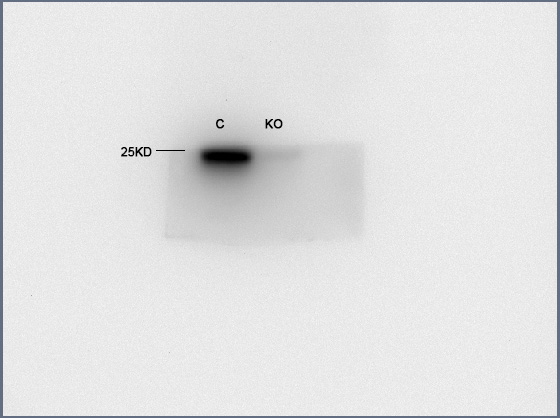

Supplement: Figure 8—source data 4. [file elife-78211-fig8-data4.zip › Figure 8-source data 4/BCLAF1/SRSF10-labeled blot.jpg]

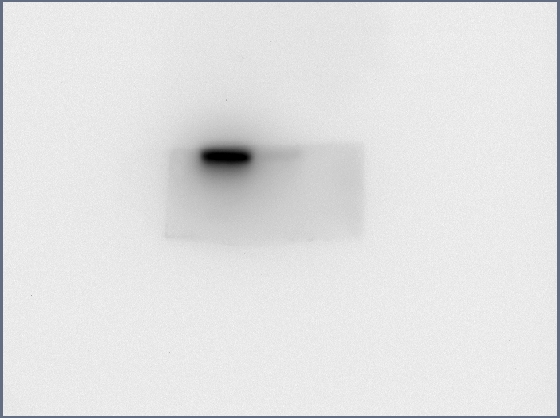

Supplement: Figure 8—source data 4. [file elife-78211-fig8-data4.zip › Figure 8-source data 4/BCLAF1/SRSF10-uncropped and unedited blot.jpg]

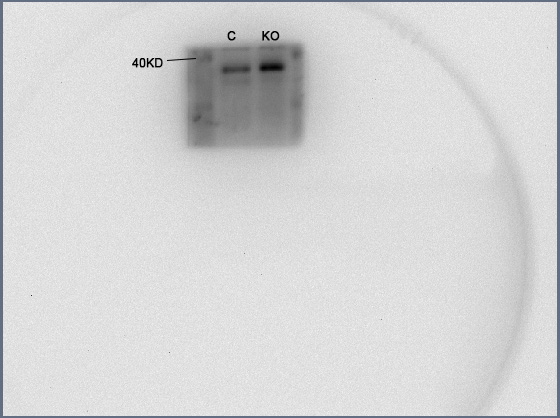

Supplement: Figure 8—source data 4. [file elife-78211-fig8-data4.zip › Figure 8-source data 4/DAZL/DAZL-labeled blot.jpg]

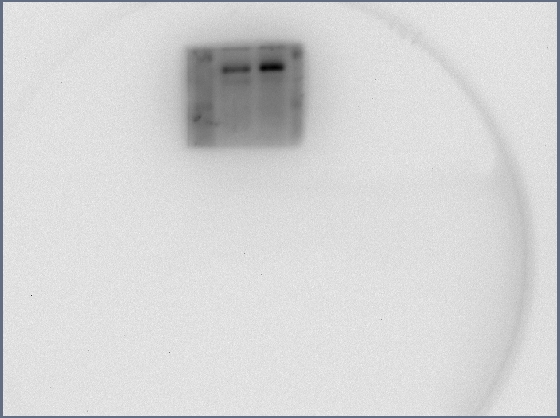

Supplement: Figure 8—source data 4. [file elife-78211-fig8-data4.zip › Figure 8-source data 4/DAZL/DAZL-uncropped and unedited blot.jpg]

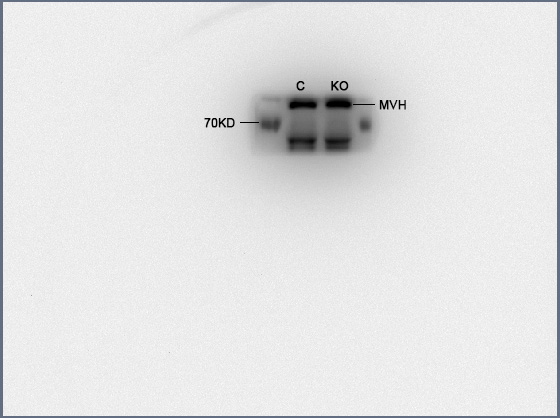

Supplement: Figure 8—source data 4. [file elife-78211-fig8-data4.zip › Figure 8-source data 4/DAZL/MVH-labeled blot.jpg]

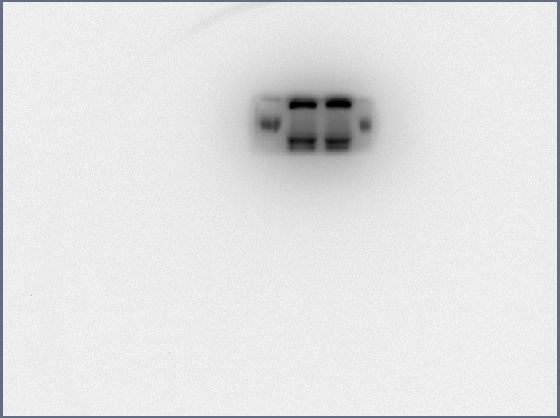

Supplement: Figure 8—source data 4. [file elife-78211-fig8-data4.zip › Figure 8-source data 4/DAZL/MVH-uncropped and unedited blot.jpg]

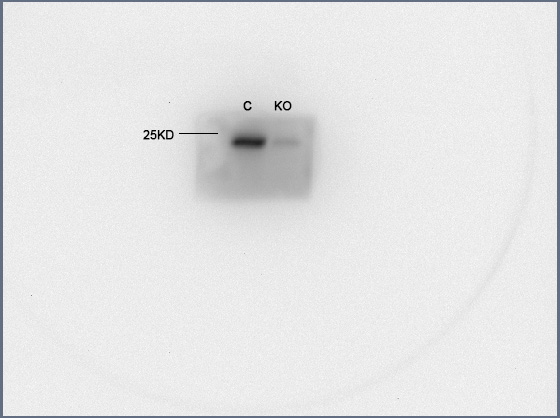

Supplement: Figure 8—source data 4. [file elife-78211-fig8-data4.zip › Figure 8-source data 4/DAZL/SRSF10-labeled blot.jpg]

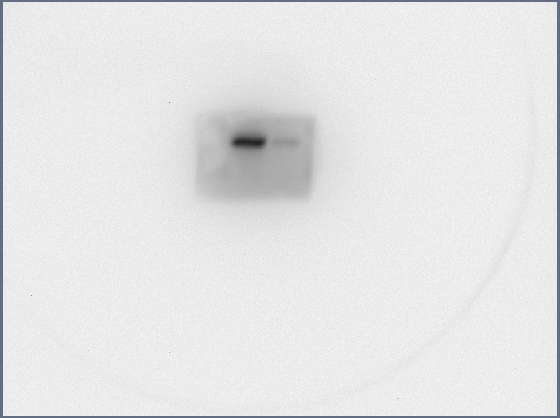

Supplement: Figure 8—source data 4. [file elife-78211-fig8-data4.zip › Figure 8-source data 4/DAZL/SRSF10-uncropped and unedited blot.jpg]

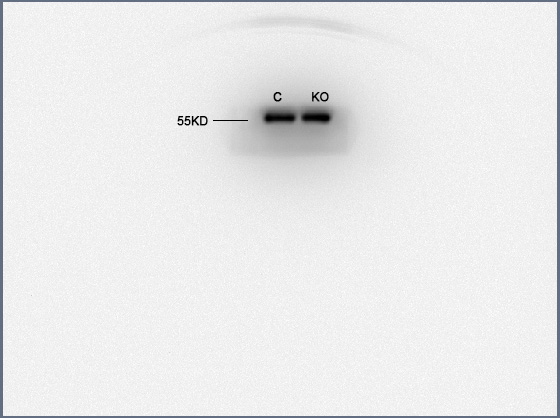

Supplement: Figure 8—source data 4. [file elife-78211-fig8-data4.zip › Figure 8-source data 4/DAZL/Tubulin-labeled blot.jpg]

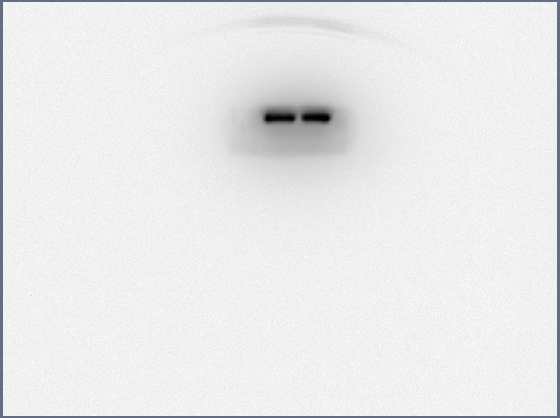

Supplement: Figure 8—source data 4. [file elife-78211-fig8-data4.zip › Figure 8-source data 4/DAZL/Tubulin-uncropped and unedited blot.jpg]

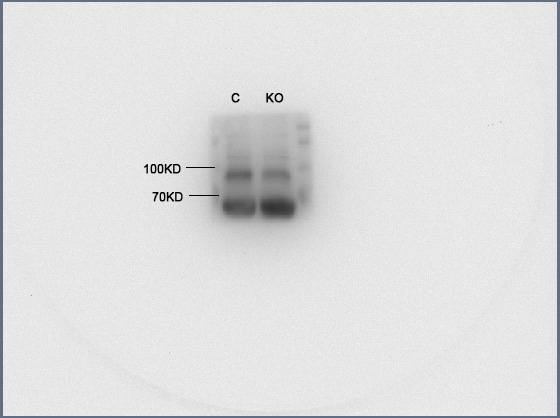

Supplement: Figure 8—source data 4. [file elife-78211-fig8-data4.zip › Figure 8-source data 4/KAT7/KAT7-labeled blot.jpg]

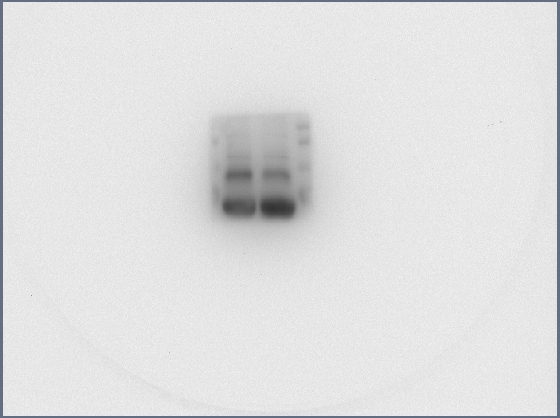

Supplement: Figure 8—source data 4. [file elife-78211-fig8-data4.zip › Figure 8-source data 4/KAT7/KAT7-uncropped and unedited blot.jpg]

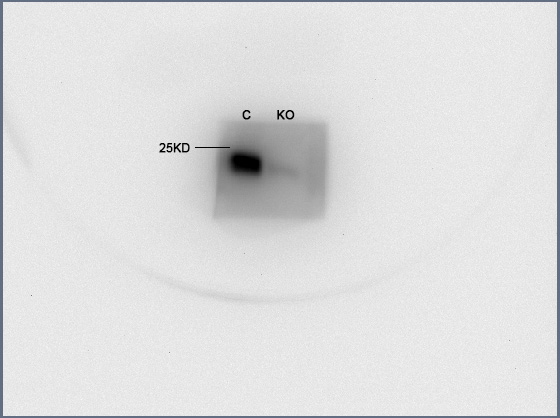

Supplement: Figure 8—source data 4. [file elife-78211-fig8-data4.zip › Figure 8-source data 4/KAT7/SRSF10-labeled blot.jpg]

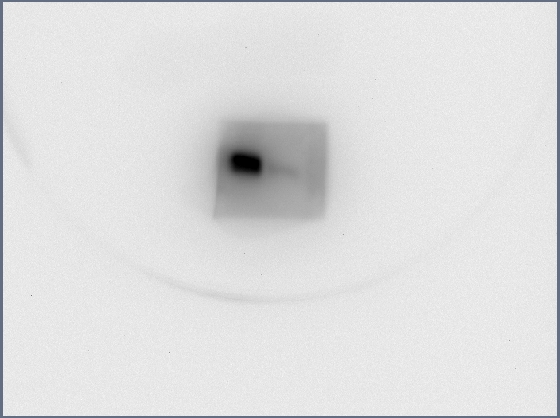

Supplement: Figure 8—source data 4. [file elife-78211-fig8-data4.zip › Figure 8-source data 4/KAT7/SRSF10-uncropped and unedited blot.jpg]

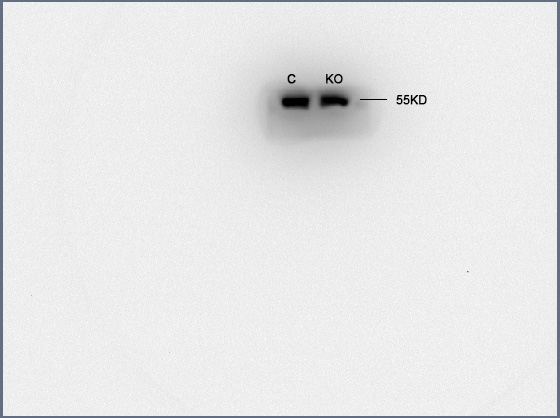

Supplement: Figure 8—source data 4. [file elife-78211-fig8-data4.zip › Figure 8-source data 4/KAT7/Tubulin-labeled blot.jpg]

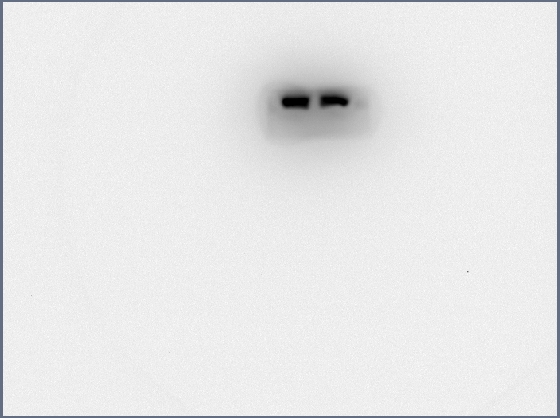

Supplement: Figure 8—source data 4. [file elife-78211-fig8-data4.zip › Figure 8-source data 4/KAT7/Tubulin-uncropped and unedited blot.jpg]

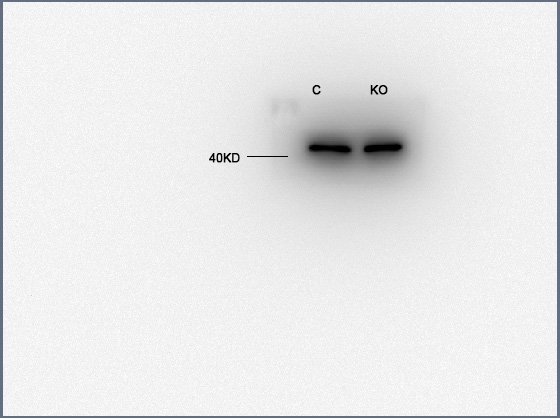

Supplement: Figure 8—source data 4. [file elife-78211-fig8-data4.zip › Figure 8-source data 4/NASP/Actin-labeled blot.jpg]

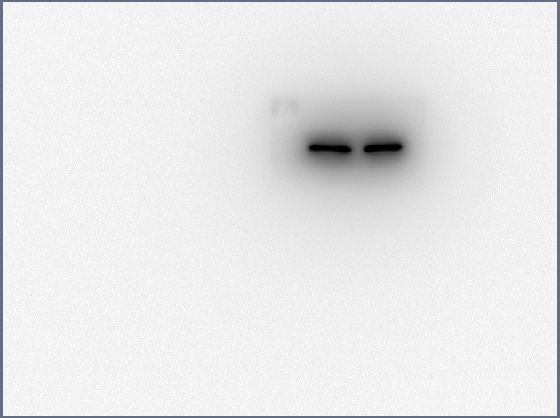

Supplement: Figure 8—source data 4. [file elife-78211-fig8-data4.zip › Figure 8-source data 4/NASP/Actin-uncropped and unedited blot.jpg]

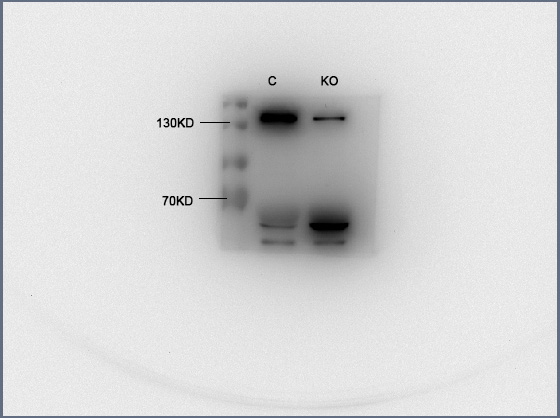

Supplement: Figure 8—source data 4. [file elife-78211-fig8-data4.zip › Figure 8-source data 4/NASP/NASP-labeled blot.jpg]

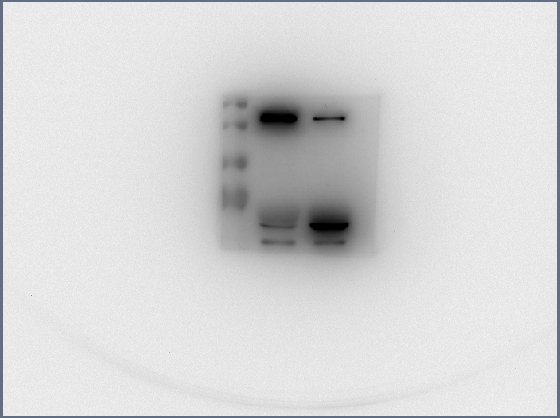

Supplement: Figure 8—source data 4. [file elife-78211-fig8-data4.zip › Figure 8-source data 4/NASP/NASP-uncropped and unedited blot.jpg]

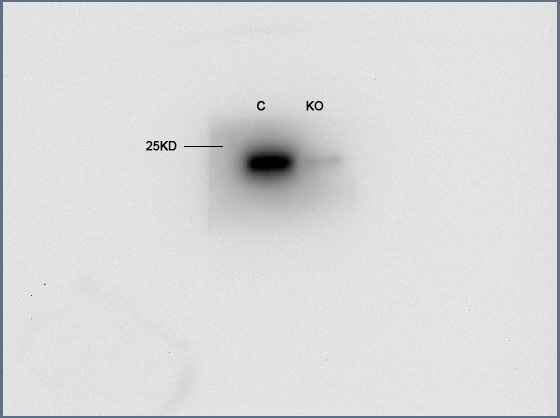

Supplement: Figure 8—source data 4. [file elife-78211-fig8-data4.zip › Figure 8-source data 4/NASP/SRSF10-labeled blot.jpg]

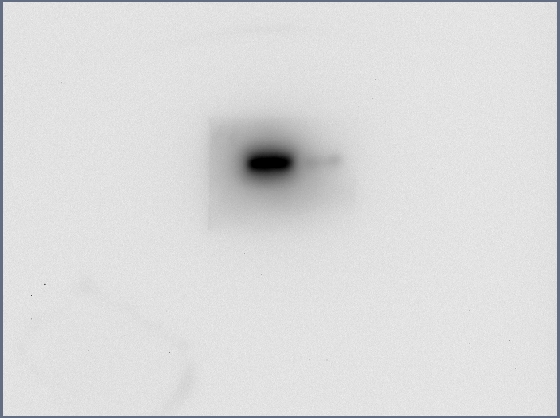

Supplement: Figure 8—source data 4. [file elife-78211-fig8-data4.zip › Figure 8-source data 4/NASP/SRSF10-uncropped and unedited blot.jpg]

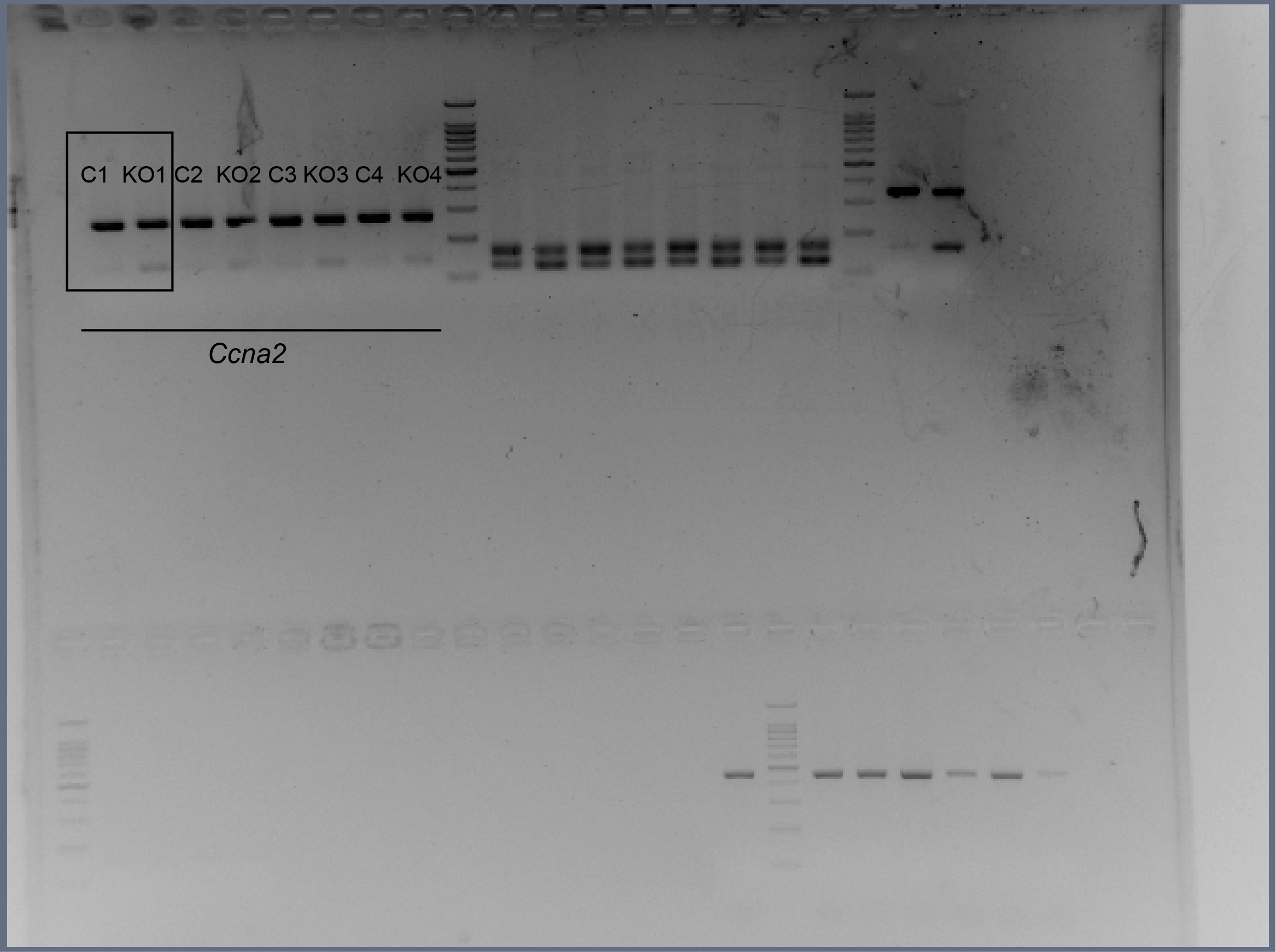

Supplement: Figure 8—figure supplement 3—source data 1. [file elife-78211-fig8-figsupp3-data1.zip › Figure 8-figure supplement 3-source data 1/Ccna2-labeled gel.jpg]

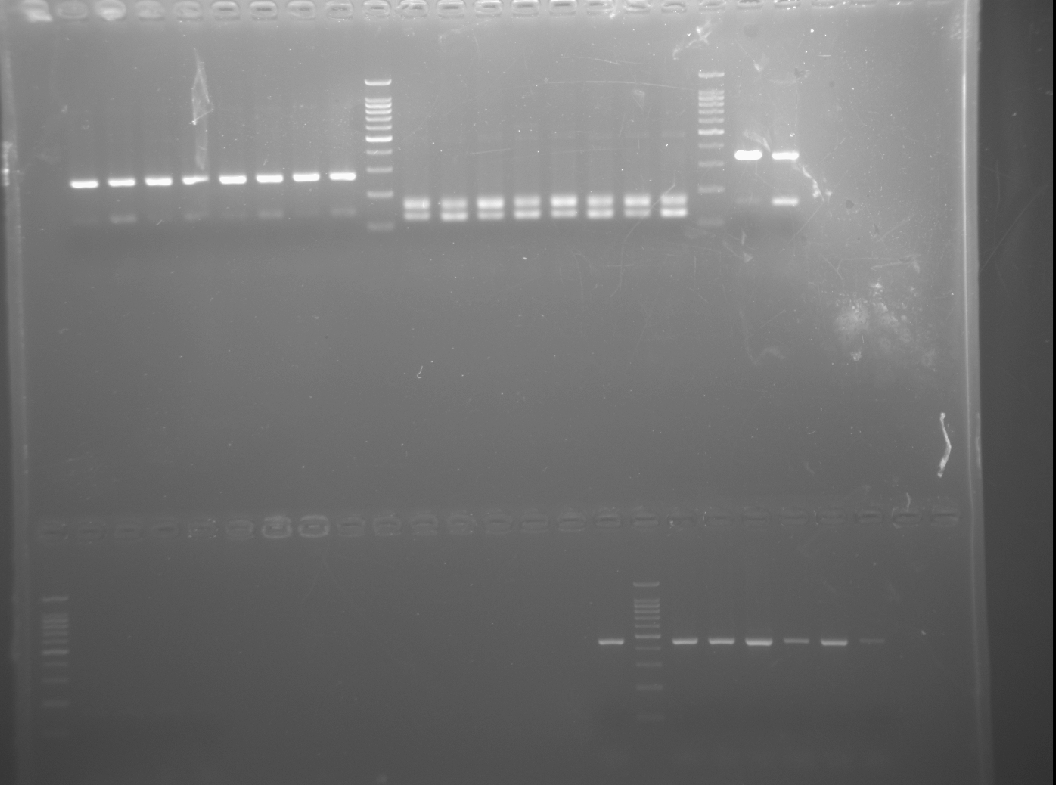

Supplement: Figure 8—figure supplement 3—source data 1. [file elife-78211-fig8-figsupp3-data1.zip › Figure 8-figure supplement 3-source data 1/Ccna2-uncropped and unedited gel.jpg]

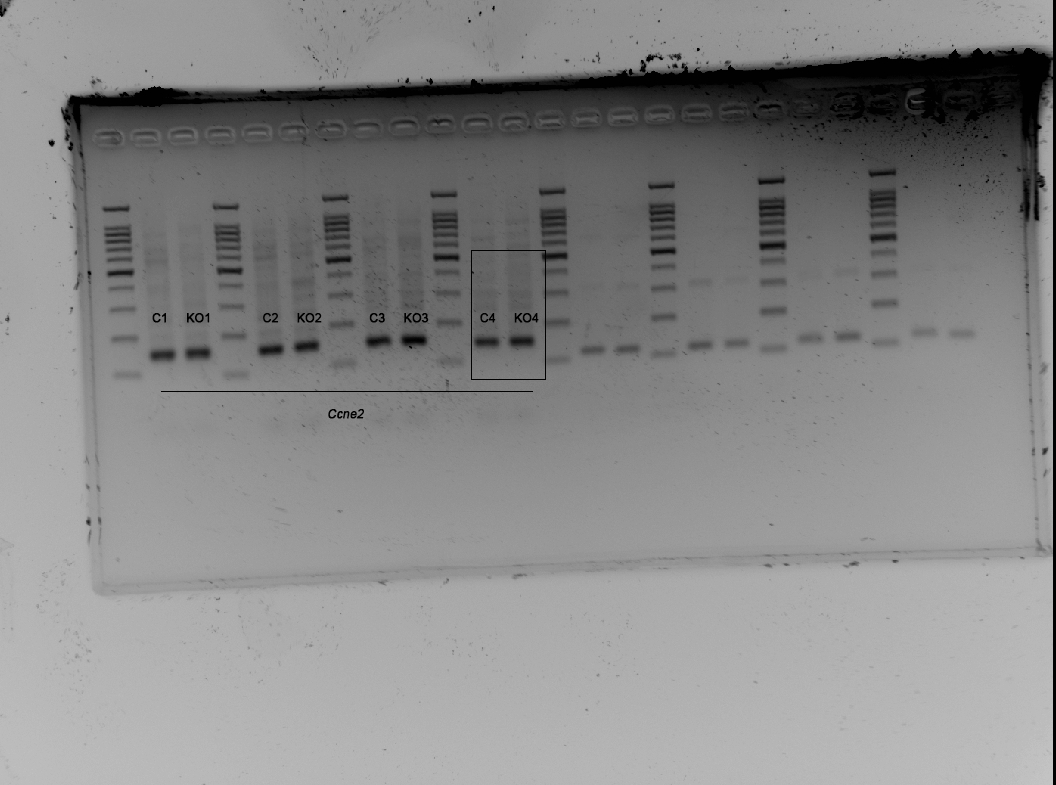

Supplement: Figure 8—figure supplement 3—source data 1. [file elife-78211-fig8-figsupp3-data1.zip › Figure 8-figure supplement 3-source data 1/ccne2-labeled gel.tif]

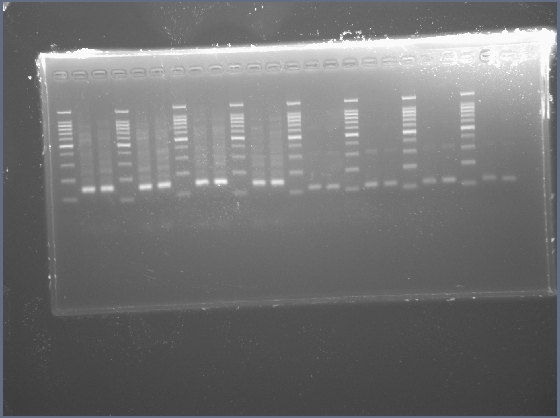

Supplement: Figure 8—figure supplement 3—source data 1. [file elife-78211-fig8-figsupp3-data1.zip › Figure 8-figure supplement 3-source data 1/Ccne2-uncropped and unedited gel.jpg]

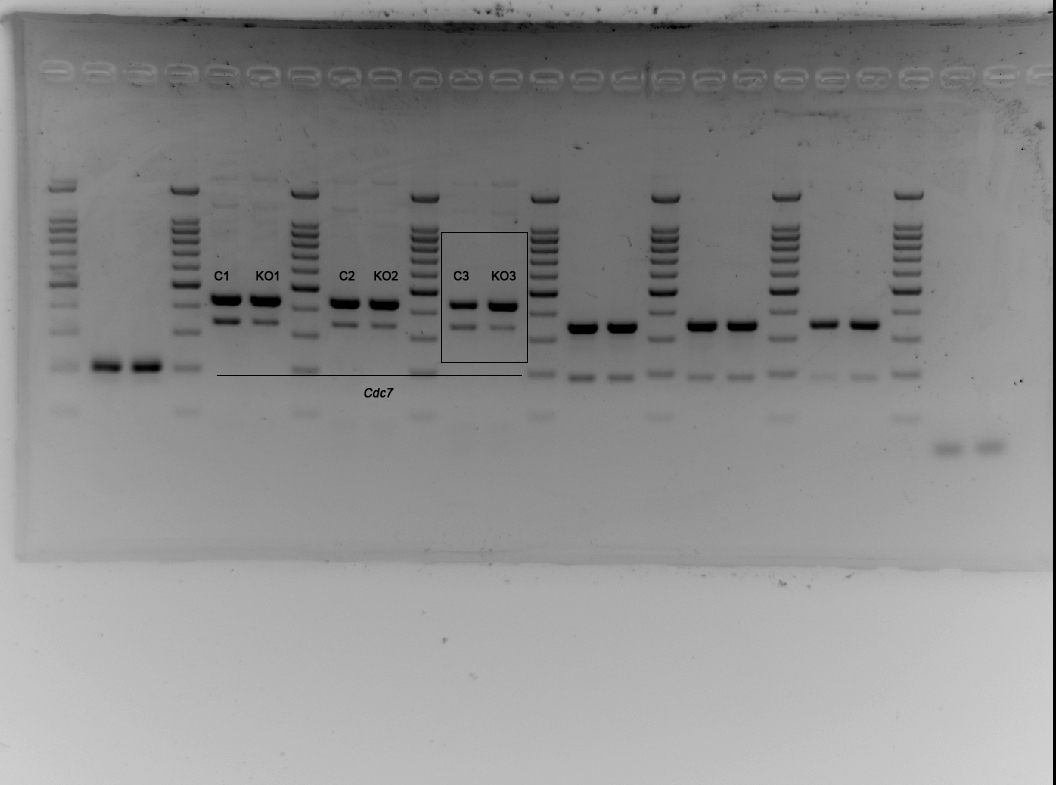

Supplement: Figure 8—figure supplement 3—source data 1. [file elife-78211-fig8-figsupp3-data1.zip › Figure 8-figure supplement 3-source data 1/Cdc7-labeled gel.tif]

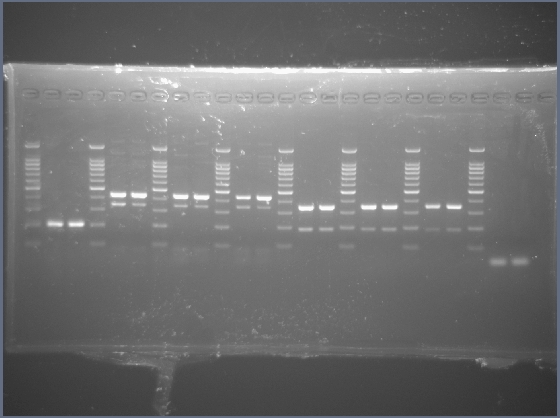

Supplement: Figure 8—figure supplement 3—source data 1. [file elife-78211-fig8-figsupp3-data1.zip › Figure 8-figure supplement 3-source data 1/Cdc7-uncropped and unedited gel.jpg]

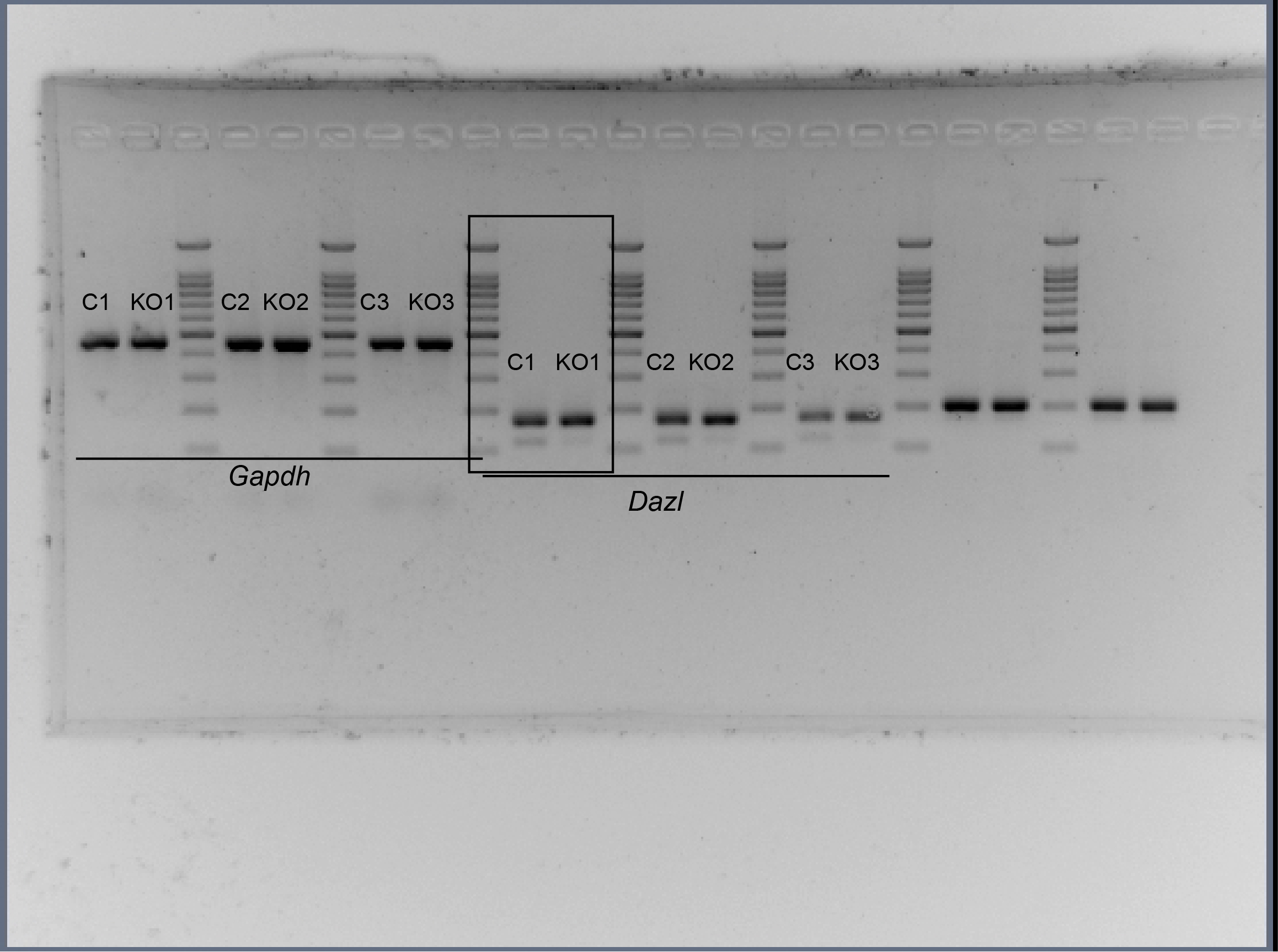

Supplement: Figure 8—figure supplement 3—source data 1. [file elife-78211-fig8-figsupp3-data1.zip › Figure 8-figure supplement 3-source data 1/Dazl-labeled gel.jpg]

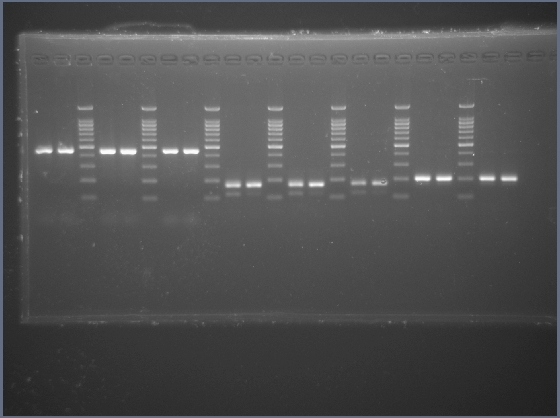

Supplement: Figure 8—figure supplement 3—source data 1. [file elife-78211-fig8-figsupp3-data1.zip › Figure 8-figure supplement 3-source data 1/Dazl-uncropped and unedited gel.jpg]

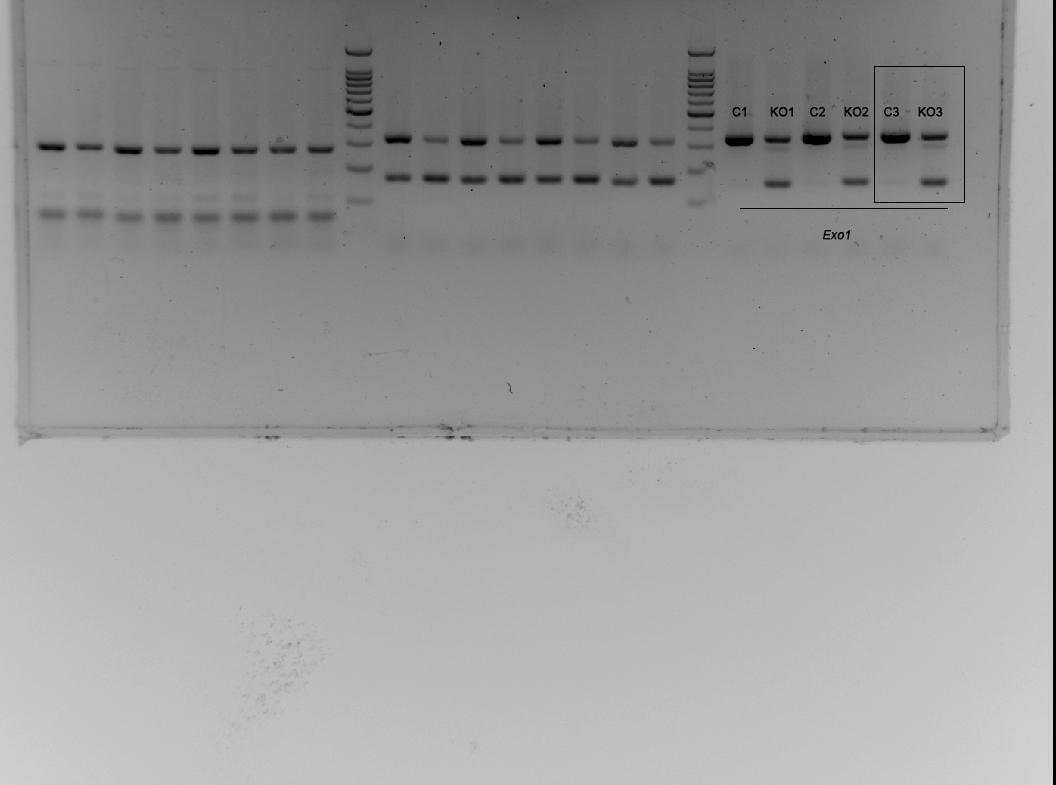

Supplement: Figure 8—figure supplement 3—source data 1. [file elife-78211-fig8-figsupp3-data1.zip › Figure 8-figure supplement 3-source data 1/Exo1-labeled gel.tif]

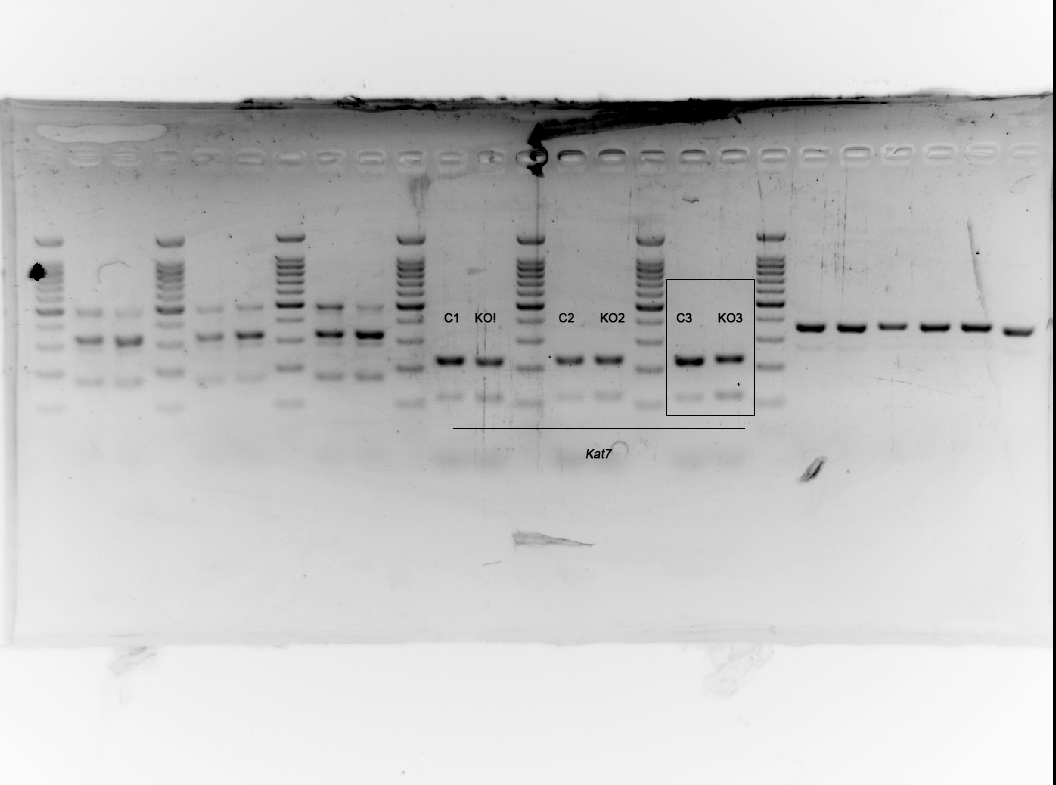

Supplement: Figure 8—figure supplement 3—source data 1. [file elife-78211-fig8-figsupp3-data1.zip › Figure 8-figure supplement 3-source data 1/Kat7-labeled gel.tif]

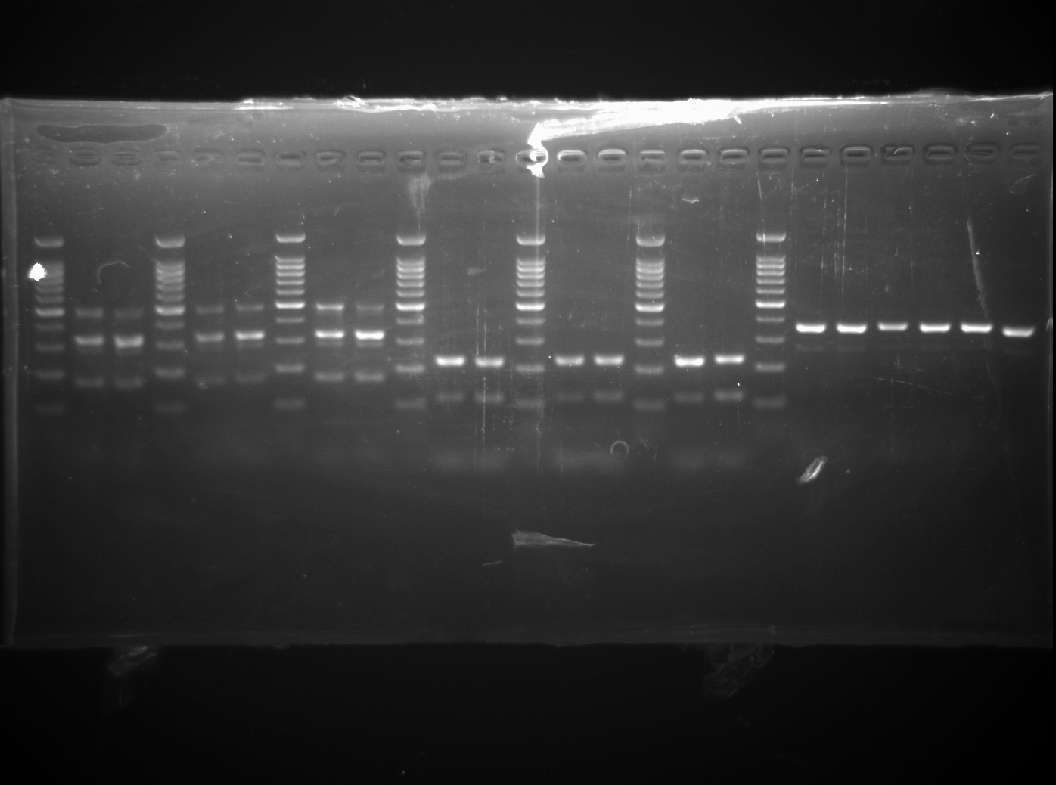

Supplement: Figure 8—figure supplement 3—source data 1. [file elife-78211-fig8-figsupp3-data1.zip › Figure 8-figure supplement 3-source data 1/Kat7-uncropped and unedited gel.jpg]

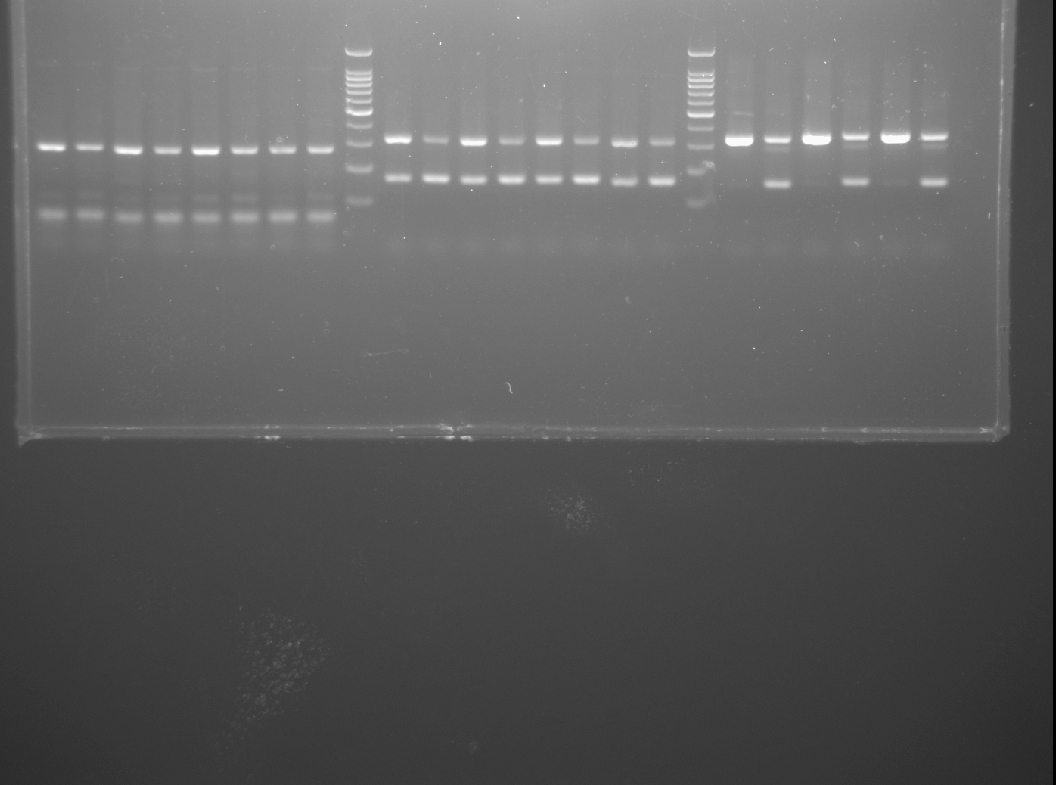

Supplement: Figure 8—figure supplement 3—source data 1. [file elife-78211-fig8-figsupp3-data1.zip › Figure 8-figure supplement 3-source data 1/Ret and Exo1-uncropped and unedited gel.jpg]

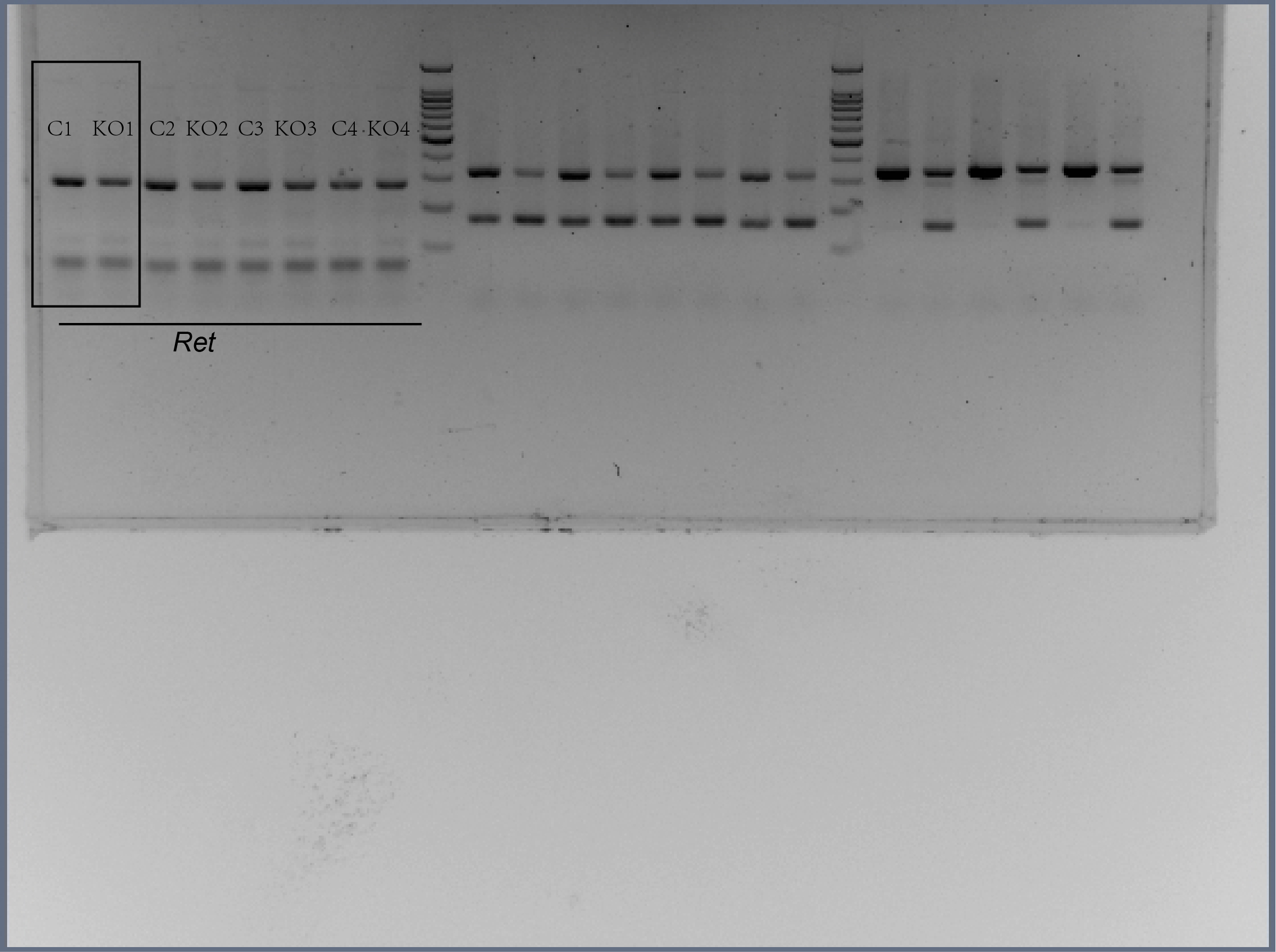

Supplement: Figure 8—figure supplement 3—source data 1. [file elife-78211-fig8-figsupp3-data1.zip › Figure 8-figure supplement 3-source data 1/Ret-labeled gel.jpg]

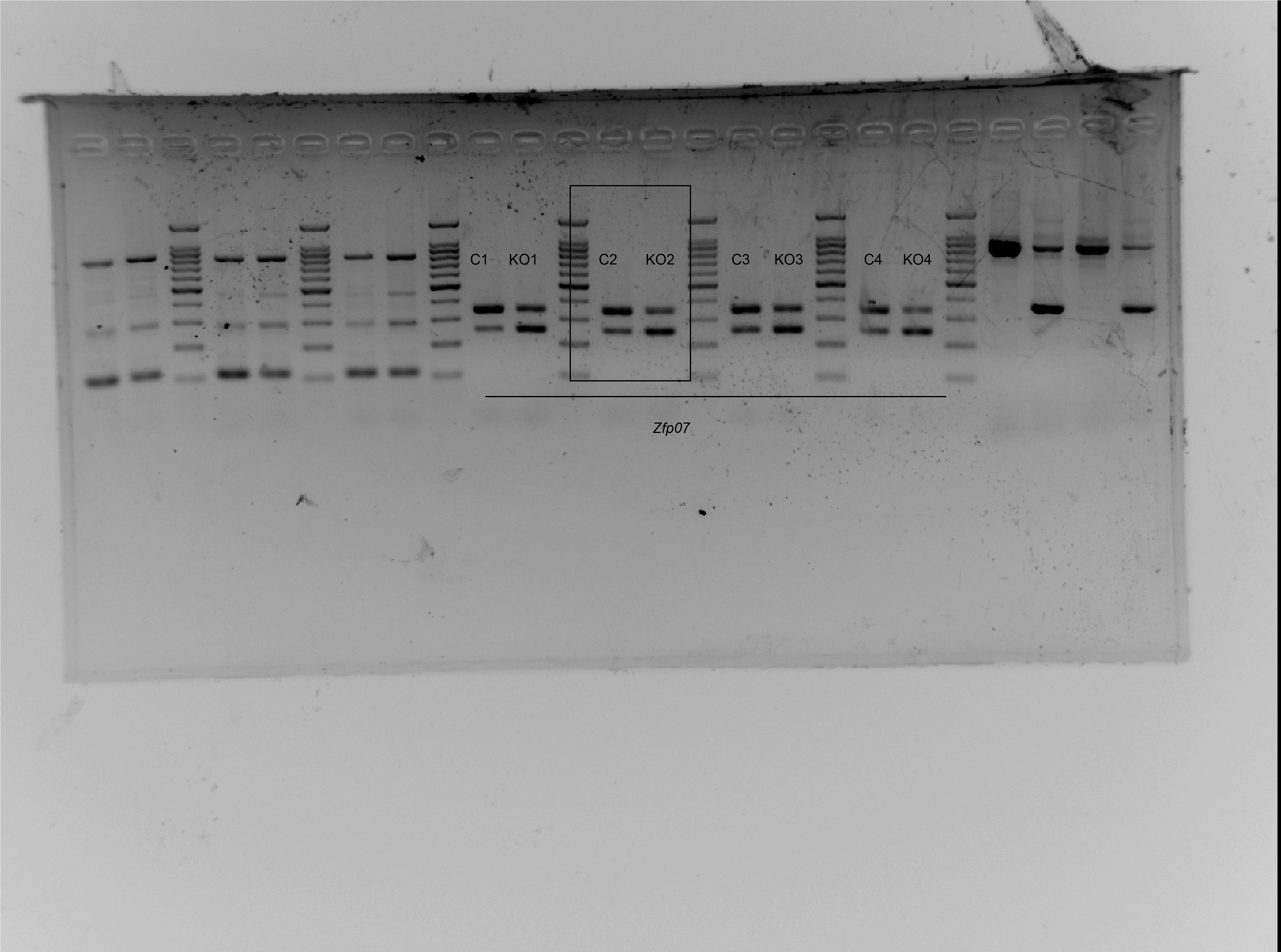

Supplement: Figure 8—figure supplement 3—source data 1. [file elife-78211-fig8-figsupp3-data1.zip › Figure 8-figure supplement 3-source data 1/Zfp207-labeled gel.jpg]

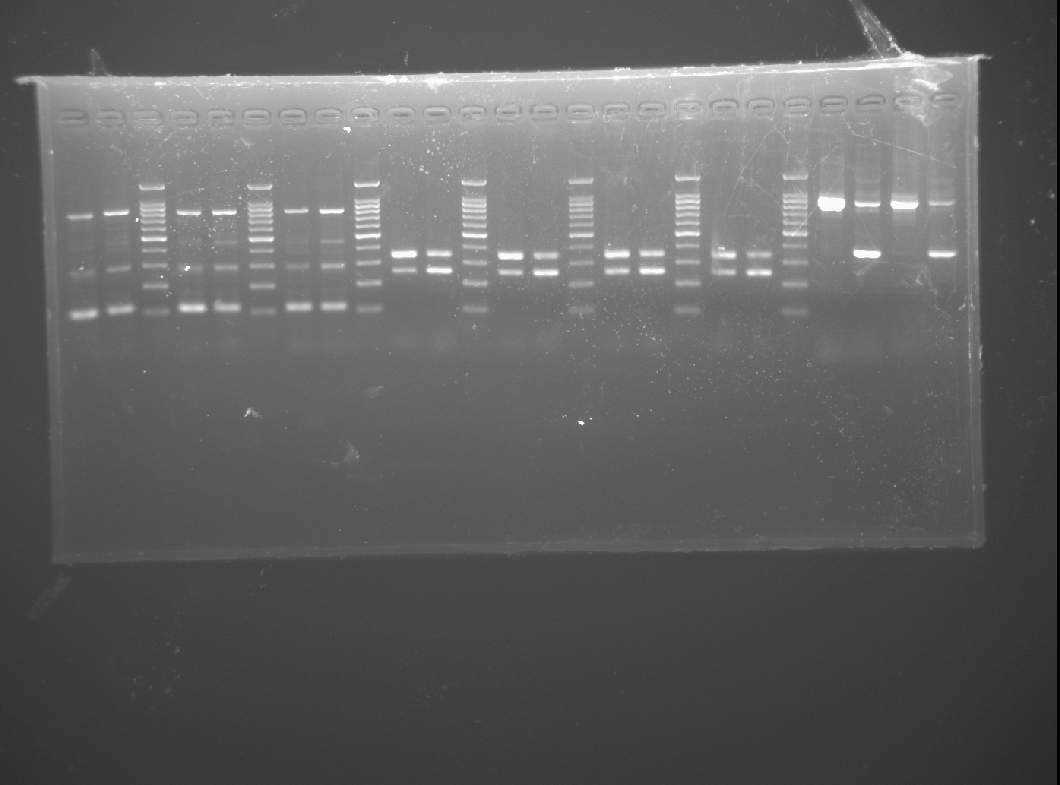

Supplement: Figure 8—figure supplement 3—source data 1. [file elife-78211-fig8-figsupp3-data1.zip › Figure 8-figure supplement 3-source data 1/Zfp207-uncropped and unedited gel.jpg]
