# Supplementary material for: SRSF10 is essential for progenitor spermatogonia expansion by regulating alternative splicing
Source: eLife. 2022 Nov 10;11:e78211. doi: 10.7554/eLife.78211 (PMC9648972; doi:10.7554/eLife.78211)
Supplement: Supplementary file 1. [file elife-78211-supp1.docx]

Table S1 Primers for quantitative PCR validation

| Gene name | Forward (5'-3') | Reverse (5'-3') |
| --- | --- | --- |
| *Bmil* | ATCCCCACTTAATGTGTGTCCT | CTTGCTGGTCTCCAAGTAACG |
| *Bcl6b* | GGCTACGTCCGAGAGTTCAC | CTTGTGCGCTCTTAGGGGT |
| *Etv5* | CAAGTCCCTTTTATGGTCCCAG | ACTCTTCAGAATCGTGAGCCA |
| *Lhx1* | TTCCTCTTGAACGTGTTGGAC | TCGGTCAGGTTGCATTTACATT |
| *Cd82* | TGGACATCATTCGCAACTACAC | GCATGGGTAAGTGGTCTTGGTA |
| *Id4* | CAGTGCGATATGAACGACTGC | GACTTTCTTGTTGGGCGGGAT |
| *GFRa1* | CACTCCTGGATTTGCTGATGT | AGTGTGCGGTACTTGGTGC |
| *Lin28a* | TGGTGTGTTCTGTATTGGGAGT | AGTTGTAGCACCTGTCTCCTTT |
| *Nanos3* | ATGGGGACTTTCAATCTTTGGAC | GTTTGCAGAATGAACATAAGCGT |
| *Neurog3* | CCAAGAGCGAGTTGGCACT | CGGGCCATAGAAGCTGTGG |
| *Sohlh1* | CGGGCCAATGAGGATTACAGA | TCCTGCGTTCTCTCTCGCT |
| *Sall4* | CCCTGGGAACTGCGATGAAG | TCAGAGAGACTAAAGAACTCGGC |
| *Zbtb16* | CTGGGACTTTGTGCGATGTG | CGGTGGAAGAGGATCTCAAACA |
| *Srsf10* | GCGTCAGATTGAAATCCAGTTCG | CGAGAGCGTCTATATCGGTCAT |
| *β-actin* | ATGCTCCCCGGGCTGTAT | CATAGGAGTCCTTCTGACCCATTC |
| *Kit* | TCATCGAGTGTGATGGGAAA | GGTGACTTGTTTCAGGCACA |
