# Supplementary material for: SRSF10 is essential for progenitor spermatogonia expansion by regulating alternative splicing
Source: eLife. 2022 Nov 10;11:e78211. doi: 10.7554/eLife.78211 (PMC9648972; doi:10.7554/eLife.78211)
Supplement: Supplementary file 2. [file elife-78211-supp2.docx]

**Supplementary file 2** Primers for validation of differential splicing changes

| Gene name | Forward (5'-3') | Reverse (5'-3') |
| --- | --- | --- |
| *Dazl* | TCCTCCTTATCCAAGTTCACCA | TCAGCTCCTGGATCAACTTCAC |
| *Gapdh* | ACCACAGTCCATGCCATCAC | TCCACCACCCTGTTGCTGTA |
| *Kit-*all | TCATCGAGTGTGATGGGAAA | GGTGACTTGTTTCAGGCACA |
| *Kit*-Full | CTTTGCATTTAAAGGTAACAACAAAG | CGACAACCTTCCATTGTACTTCATA |
| *Kit-*short | TGCATTTAAAGAGCAAATCCAGG | CGACAACCTTCCATTGTACTTCATA |
| *Cdc7* | ATGCCTTGGTGGACTTCGG | GTTTCCCTCATCACGCTGTTC |
| *Kat7* | CATAAGCTACCGCAGCTACTGG | GGCCTCTTTGGCTATCCACTC |
| *Cenph* | CAGTTGCACTTCGGGATAACA | GTCCTCTGCCCAGTTGGTTT |
| *Rif1* | CAAGCAGGATTGGCAGATGA | GGAGAACGGATAGGCAGAGTTT |
| *Nasp* | GGAGTGTTGGGAAATGCCTTAG | TTCCTCTGAGCCTTCAGTTTCTT |
| *Ccne2* | AGCTGAGCCGAGCTGTGG | GGCCTGAATTATCTGGGTT |
| *Ret* | TGGTCCTTTGGAGTGCTGCT | ATCGCTGGGAGCCAAGAC |
| *Zfp207* | TTCCTCCAATGACTCAAG | CTGTAGACTGTGTATAAGC |
| *Bclaf1* | TGGGTCTGGTTCTGTTGGAAAT | AGCAAGCAGCCTGTCTTTAGTC |
| *Exo1* | CCCACTCAAGAAGCCACA | GGGCAGGACTTGGACATT |
| *Mcm10* | GAAGAGGCTGGCAGTGGAGA | GGAACAGCTTGAGGTGAGGGT |
| *Sycp1* | TGCTAATTCTGGCAGTTG | GCACAGGTTTCCTTGAGT |
| *Ccna2* | ACTGAGTTTGATAGATGCTGAC | TCTGGTGGGTTGAGAAGA |
